# P-2102. Implementation of a Virtual Pharmacy-led Travel Clinic Pilot Program at Cleveland Clinic

**DOI:** 10.1093/ofid/ofaf695.2266

**Published:** 2026-01-11

**Authors:** Jona Banzon, Pooja Cerrato, Bethany Lehman, Patricia Bartley, Steven Mawhorter, Andrea Pallotta, Steven M Gordon

**Affiliations:** Cleveland Clinic, Cleveland, Ohio; Cleveland Clinic, Cleveland, Ohio; Cleveland Clinic, Cleveland, Ohio; Cleveland Clinic, Cleveland, Ohio; Cleveland Clinic, Cleveland, Ohio; Cleveland Clinic, Cleveland, Ohio; Cleveland Clinic Foundation, Cleveland, OH

## Abstract

**Background:**

The resurgence of international travel in the post-COVID-19 era has led to an increase in demand for travel medicine services. In response, a virtual travel clinic led by a pharmacist certified in travel health, working under a collaborative practice agreement with the Cleveland Clinic Infectious Disease department, was launched in 2023. Five community pharmacies served as the effector arm for vaccine administration and prescriptions.

**Figure:**
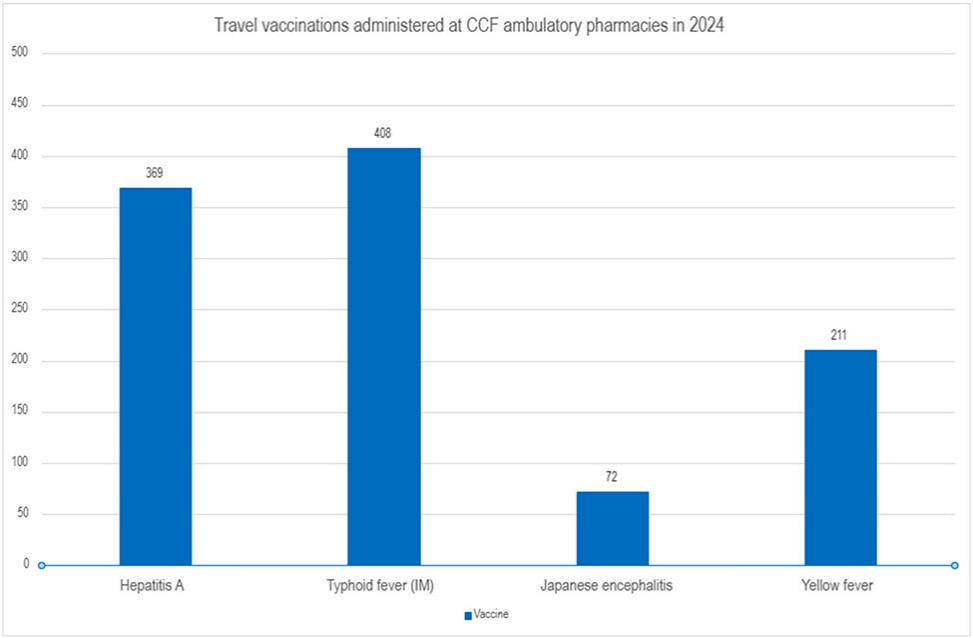

**Figure:**
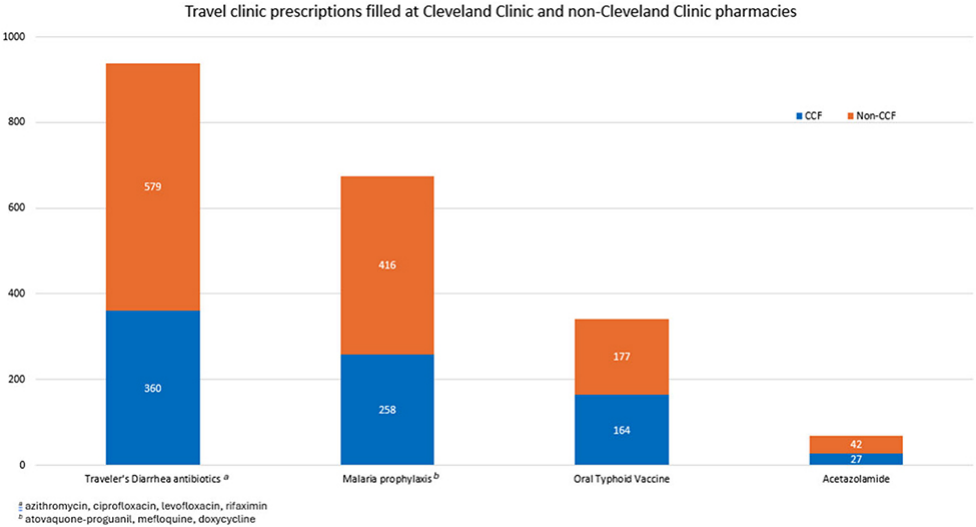

**Methods:**

Patients seen at Cleveland Clinic from January 1 to December 31, 2024 through the pharmacy-led travel clinic were included. Electronic medical records were reviewed for patient demographics, travel plans, recommendations for vaccines and prescription drugs, vaccine administration and prescription drug pharmacy fills. Pregnant and immunosuppressed patients were excluded from this clinic and seen by a physician. Children under 7 years old received vaccinations at pediatric clinics, as per Ohio regulations.

**Results:**

There were 1,088 pharmacy-led travel clinic encounters conducted in 2024. 1,076 (99%) of these were conducted virtually. In June 2024, a virtual shared medical appointment format was added to increase capacity, and 55 (5%) patients were seen in these shared visits from June to December 2024. The median age was 47 (range, 0-85). Patients were primarily referred by Cleveland Clinic practitioners, while a minority were referred by occupational health. 1060 representative travel vaccinations consisting of hepatitis A, intramuscular typhoid, Japanese encephalitis B, and yellow fever vaccines, were administered at the community pharmacy sites, with typhoid being the most common (Figure 1). Travel-related medications, including oral typhoid vaccine, were filled at our institution pharmacies 41% of the time.

**Conclusion:**

Collaborative practice agreements with pharmacy play an essential role in providing valuable services, especially in the era of physician shortages. In particular, the focus on preventative care and education involved in travel medicine makes this well-suited for such a partnership. A virtual platform provides a scalable model that increases patient access and convenience, and can lead to improved uptake of recommendations. Further data on patient satisfaction and health outcomes is needed.

**Disclosures:**

Bethany Lehman, DO, Insmed: Advisor/Consultant|Merk: Honoraria

